# Watershed boundaries and geographic isolation: patterns of diversification in cutthroat trout from western North America

**DOI:** 10.1186/1471-2148-12-38

**Published:** 2012-03-19

**Authors:** Janet L Loxterman, Ernest R Keeley

**Affiliations:** 1 Department of Biological Sciences, Idaho State University, Stop 8007, Pocatello, ID, USA 83209

## Abstract

**Background:**

For wide-ranging species, intraspecific variation can occur as a result of reproductive isolation from local adaptive differences or from physical barriers to movement. Cutthroat trout (*Oncorhynchus clarkii*), a widely distributed fish species from North America, has been divided into numerous putative subspecies largely based on its isolation in different watersheds. In this study, we examined mtDNA sequence variation of cutthroat trout to determine the major phylogenetic lineages of this polytypic species. We use these data as a means of testing whether geographic isolation by watershed boundaries can be a primary factor organizing intraspecific diversification.

**Results:**

We collected cutthroat trout from locations spanning almost the entire geographic range of this species and included samples from all major subspecies of cutthroat trout. Based on our analyses, we reveal eight major lineages of cutthroat trout, six of which correspond to subspecific taxonomy commonly used to describe intraspecific variation in this species. The Bonneville cutthroat trout (*O. c. utah*) and Yellowstone cutthroat trout (*O. c. bouvieri*) did not form separate monophyletic lineages, but instead formed an intermixed clade. We also document the geographic distribution of a Great Basin lineage of cutthroat trout; a group typically defined as Bonneville cutthroat trout, but it appears more closely related to the Colorado River lineage of cutthroat trout.

**Conclusion:**

Our study indicates that watershed boundaries can be an organizing factor isolating genetic diversity in fishes; however, historical connections between watersheds can also influence the template of isolation. Widely distributed species, like cutthroat trout, offer an opportunity to assess where historic watershed connections may have existed, and help explain the current distribution of biological diversity across a landscape.

## Background

Species with wide geographic ranges often exhibit significant morphological, behavioral, or genetic variability across their range [1]. Such differences have been attributed to local adaptation and genetic drift as a result of isolation of populations by limited gene flow [2]. Although the importance of within-species diversity has been increasingly recognized by subspecific taxonomy, distinct population segments, and evolutionarily significant units [3,4], most studies have focused on understanding how phenotypic variability can influence the distributional limits of a species [5]. Intraspecific variability can represent significant adaptive potential allowing a species to occupy a broad range of ecological conditions or adapt to changing environmental conditions [6]. In addition to understanding the potential importance of phenotypic limits to a species distribution, past studies have also tried to identify physiographic features that may facilitate, organize, or constrain diversification of species that are distributed over large geographic areas [7,8].

Across a landscape, geographic isolation may arise as a consequence of barriers to movement from mountain building, glacial events, continental drift or a combination of factors [9]. While wide-ranging terrestrial species can exhibit geographic isolation and population structuring [10,11], aquatic species and freshwater fishes, in particular, often exhibit strong population structure likely resulting from their confinement to the network or 'ribbons' of water that flow across the landscape. The height of land that separates watershed boundaries and the relatively common occurrence of movement barriers within watersheds often isolate fish populations allowing for significant genetic differentiation [12-15].

In addition to the slow and continuous erosive changes to watersheds, major drainage patterns can be changed by events such as volcanic or glacial flows, or by climatic changes in precipitation that can connect or isolate watersheds [16-18]. Newly created watershed connections are thought to be important influences shaping community composition for organisms like fishes that are obligatorily confined to the network of streams and lakes by allowing species that were previously isolated to expand their geographic range [8]. In contrast, populations of the same species that become isolated by loss of connectivity may diversify into new forms representing early stages of speciation [7].

**Figure 1:**
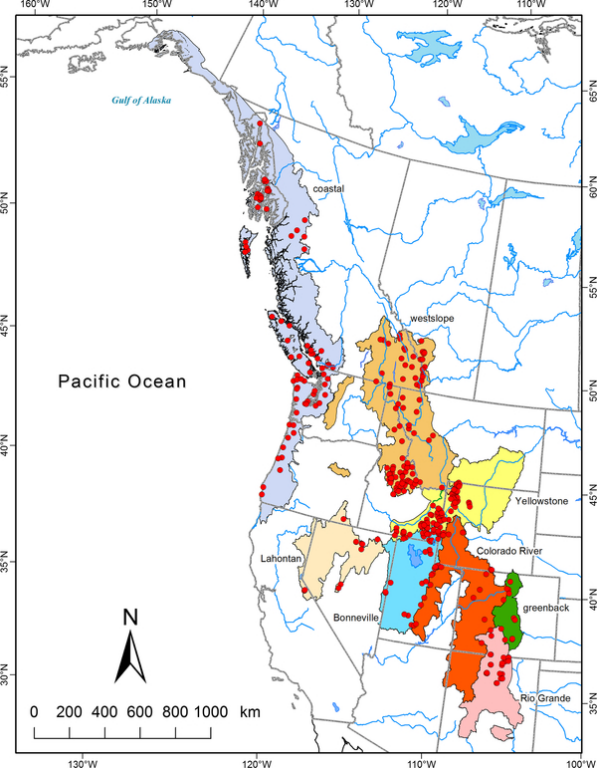
**Historical range of cutthroat trout and sampling localities**. Sampling locations of cutthroat trout from western North America (red dots) within native ranges of eight major subspecies of cutthroat trout (colored polygons). Historical distributions are based on Behnke [24] and McPhail [64].

The cutthroat trout (*Oncorhynchus clarkii; *Teleostei: Salmonidae) is a widely distributed fish species native to western North America that occurs along a north-south axis from Alaska to New Mexico (Figure 1). Populations of cutthroat trout are found in very diverse habitats ranging from coastal temperate rainforest watersheds to desert lakes and streams [19,20]. Perhaps not surprisingly, cutthroat trout exhibit dramatic morphological and life history diversity across these contrasting ecological conditions and have been divided into as many as 14 different subspecies to account for much of this diversity [19,21]. Despite the range of ecological diversity encompassed by cutthroat trout, different subspecies are primarily organized by major watershed boundaries that are often contiguous to each other and separated only by the height of land between adjacent areas (Figure 1). In addition to diversification created by isolation in different watersheds, cutthroat trout also occupy northern areas that were covered in ice during the last glacial maximum and re-invaded from southern ice-free watersheds by connections during pluvial periods. In western North America, the last period of glaciation reached a maximum about 18 000 years ago and covered much of present day Canada as well as northern portions of adjoining areas in the United States [22]. Previous comparisons of species occupying formerly glaciated and non-glaciated regions often reveal lower levels of genetic diversification in formerly glaciated portions of their range [17]; indicating the importance of climatic changes in the environment for species diversification.

Given their range and distribution, cutthroat trout populations offer an excellent opportunity to examine how geographic features such as major watershed boundaries can organize and shape genetic diversification in wide-ranging fish species. Similarly, their distribution across geographic regions that have experienced significant changes in climatic conditions also offers an opportunity to understand how physiographic changes in climate can influence the template of diversification by watershed isolation.

In this study we examine how the diversity and evolutionary history of a widely distributed fish species can be organized by primary geographic features such as watershed boundaries and how such organization may also be influenced by historic events that have connected watersheds but are no longer visible on the landscape. Although intraspecific taxonomic categories are often used in the management of cutthroat trout populations [23] there is remarkably little comparative data to assess whether subspecific differences capture or reliably organize the diversity present in this widely distributed species. In this study we sampled cutthroat trout from throughout their geographic range to compile a comprehensive database of mtDNA sequence data as a means of documenting the evolutionary history of cutthroat trout. By doing so, we test how well geographic features and past subspecific divisions of cutthroat trout represent the diversity within this species and how past connections between watersheds may have influenced this organization.

## Methods

### Study sites

We sampled extant populations of cutthroat trout from 311 locations representing the majority of their geographic range (Figure 1, Additional file 1: Table S1) to compare mitochondrial (mtDNA) sequence divergence at the NADH dehydrogenase subunit 2 gene (ND2) and determine major phylogenetic branching among groups. Past taxonomic evaluations of cutthroat trout have listed up to 14 different subspecies of cutthroat trout [20,24]. In our study we included samples from eight major subspecies that are commonly recognized, including: Bonneville cutthroat trout (*Oncorhynchus clarkii utah*), coastal cutthroat trout (*O. c. clarkii*), Colorado River cutthroat trout (*O. c. pleuriticus*), greenback cutthroat trout (*O. c. stomias*), Lahontan cutthroat trout (*O. c. henshawi*), Rio Grande cutthroat trout (*O. c. virginalis*), westslope cutthroat trout (*O. c. lewisi*), and Yellowstone cutthroat trout (*O. c. bouvieri*). We also included samples of cutthroat trout from populations considered by some to be separate subspecies of cutthroat trout, including willow/Whitehorse (*O. c. ssp*.), Paiute (*O.c. seleneris*) Humboldt (*O. c. humboldtensis*), and fine-spotted Snake River (*O. c. behnkei*) cutthroat trout [19,20,25]. Two other subspecies, the Alvord cutthroat trout (*O. c. alvordensis*) and yellowfin cutthroat trout (*O. c. macdonaldi*), are thought to be extinct [20]. Because we were interested in examining geographic patterns of natural variation in sequence divergence, we sampled populations that are believed to be representative of native populations. In addition, we sampled nine rainbow trout (*O. mykiss*) populations to use as an outgroup for our phylogenetic analyses (Additional file 1: Table S1).

To collect cutthroat trout from as wide a geographic range as possible, we used a variety of sampling techniques. In streams, we used a backpack or boat-mounted electroshocker or angled for fish. For lake populations, we sampled fish by angling, or by using multi-panel monofilament gillnets, minnow traps, or trap nets. Captured fish were held in a 20 L container with fresh water, and a 3-5 mm fin clip was taken from each fish and stored in a uniquely numbered vial with 95% ethanol. In four locations, supplementary genetic samples were provided by researchers using similar sampling methods.

### GenBank sequences

In addition to the 311 locations we physically sampled or were sampled for us, we also searched the National Center for Biotechnology Information's GenBank database http://www.ncbi.nlm.nih.gov/genbank/ for ND2 sequences of cutthroat trout. We included samples from this database in geographic comparisons whenever we could identify locations either in the GenBank database or from corresponding publications that provided location information (Additional file 2: Table S2).

### DNA extraction and mtDNA sequencing

DNA was extracted from fin clips using the ZR Genomic DNA tissue extraction kit (Zymo Research) following the manufacturer's protocol. Mitochondrial DNA variation was analyzed using the entire ND2 gene and was amplified by polymerase chain reaction (PCR) using the sequencing primers NDintF6 (5' TAAGCTTTCGGGCCCATACC 3') and NDVarR (5'GCT TTG AAG GCT CTT GGT CT 3') [26,27]. Our PCR reactions were performed in 25 μl volumes using 8 μL of 2X ReddyMix PCR Master Mix (ABgene), 1 μL (10 mM) of each primer, and 2 μL of genomic DNA. The thermal profile included an initial 94°C denaturation followed by 35 cycles at 94° for 30 s, annealing at 58°C for 45 s, and extension at 72°C for 75 s, with a final extension at 72°C for 10 min. PCR products were submitted to the University of Washington High-throughput genomics unit for purification and DNA sequencing.

### Sequence data analysis

We edited and aligned the sequences using Sequencher v4.9 (Gene Codes Corporation) and the online version of Muscle [28]. Unique sequences were submitted to GenBank with accession numbers as reported in Additional file 1: Table S1. To illustrate the relationship between unique cutthroat trout haplotypes, we constructed a minimum spanning network using representatives of each unique ND2 haplotype and the program Arlequin v3.5 [29]. Mitochondrial DNA polymorphism was estimated as haplotype (*h*) and nucleotide (π) diversity, as well as the percent sequence divergence between groups using DNAsp v5 [30] and Mega v5.01 [31] software.

### Phylogenetic analyses

For our phylogenetic analyses, we included one sequence representing each unique haplotype, as well as rainbow trout sequences, which were used as an outgroup. Phylogenies were estimated by maximum likelihood (ML) and Bayesian analyses. For our ML analysis, we used the Tamura-Nei substitution model with invariant sites and gamma-distributed rates among sites based on jModeltest [32] results and generated 1000 bootstrap replicates as implemented in the program Phyml v3.0 [33]. Our Bayesian analysis was conducted using MrBayes v3.1.2 [34,35] with the GTR + *I *+ *Γ *model based on MrModeltest [36]. The analyses included two independent runs for 5.5 million generations each and were sampled every 100^th ^generation. The first 25% of samples were discarded as "burn-in" to ensure sampling from a stationary posterior distribution.

We used the primary lineages derived in phylogenetic analyses to compare the sequence diversity within and among groups and estimate their divergence times. The proportion of diversity within and among primary phylogenetic groups was estimated using an AMOVA as implemented in Arlequin v3.5. We estimated divergence times between primary cutthroat trout lineages by first testing for equal evolutionary rates between lineages using Tajima's relative rate test [37] and then calibrating and linearizing our phylogenetic tree using Mega 5.01 software. We applied the estimated divergence time of 6 million years ago (mya) to the node between rainbow trout and cutthroat trout to calibrate our tree. This divergence time is based on the combination of fossil data and DNA sequence data [38] and is within the range of proposed estimates of divergence between rainbow trout and cutthroat trout [39].

## Results

**Figure 2:**
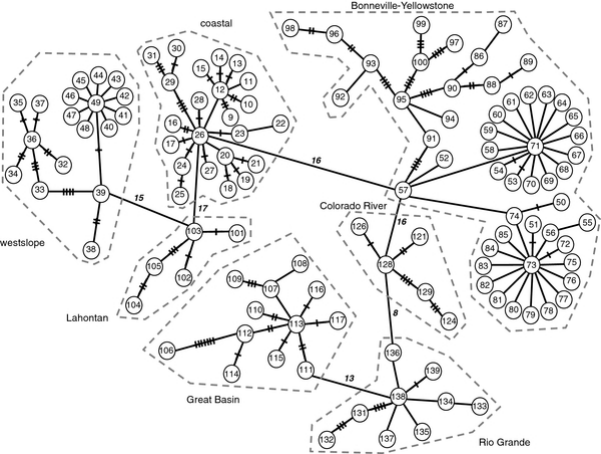
**ND2 haplotype network for cutthroat trout**. Minimum-spanning network of mtDNA haplotypes at the ND2 gene in cutthroat trout. Numbers within circles refer to haplotypes listed in Additional file 1: Table S1 and Additional file 2: Table S2. Hash marks (up to eight) indicate the number of mutational changes. One step is indicated by a line with no hash marks and numbers adjacent to lines indicate more than eight mutational changes. Only cutthroat trout haplotypes with 1050 bp are included in the network.

Nucleotide sequences were generated for ND2 (1050 bp) for 384 trout representing 311 populations and 102 different haplotypes. We also compiled sequence data from an additional 95 cutthroat trout samples (Yellowstone cutthroat trout [n = 71], Lahontan cutthroat trout [n = 4], Rio Grande cutthroat trout [n = 10], Colorado River cutthroat trout [n = 6], and greenback cutthroat trout [n = 4]) deposited in the GenBank database [27,40,41]; providing an additional 38 haplotypes. Yellowstone cutthroat trout ND2 sequences from GenBank were 1050 bp, all other subspecies had ND2 sequences of 889 bp in length. Hence, when we combined our sequences with those from Genbank (both 889 bp and 1050 bp), a total of 140 different haplotypes of cutthroat trout were available for comparison (Table 1). A minimum spanning network of all haplotypes with complete sequences (1050 bp) revealed seven main clusters of haplotypes that corresponded to some of the subspecific classifications of cutthroat trout (Figure 2). Haplotypes from the Bonneville and Yellowstone cutthroat trout did not form distinct clusters, but were intermixed. In contrast, haplotypes from populations of coastal, Colorado River, Lahontan, Rio Grande, and westslope cutthroat trout formed separate clusters. Interestingly, we also identified an additional cluster that included populations from within the Bonneville and Yellowstone cutthroat trout distribution, which we refer to as the Great Basin cutthroat trout lineage because of its geographic distribution within the central portion of the Great Basin region of North America (see below). The number of mutational steps separating each major group ranged from 8 to 17 (Figure 2).

**Table 1:** Main groupings or taxa used in phylogenetic comparisons of cutthroat trout and rainbow trout, sample size (N), number of unique haplotypes, haplotype diversity (*h*) and nucleotide diversity (π)

Taxa/Clade	N	haplotypes	*h*	π
rainbow trout	18	8	0.88	0.007
coastal cutthroat trout	83	23	0.86	0.003
westslope cutthroat trout	94	18	0.72	0.004
Bonneville-Yellowstone cutthroat trout	166	51	0.92	0.007
Lahontan cutthroat trout	20	5	0.80	0.001
greenback cutthroat trout	4	4	1.00	0.009
Great Basin cutthroat trout	38	12	0.89	0.003
Colorado River cutthroat trout	27	9	0.83	0.002
Rio Grande cutthroat trout	24	10	0.89	0.002
Total	474	140	0.97	0.022

### Primary phylogenetic lineages

**Figure 3:**
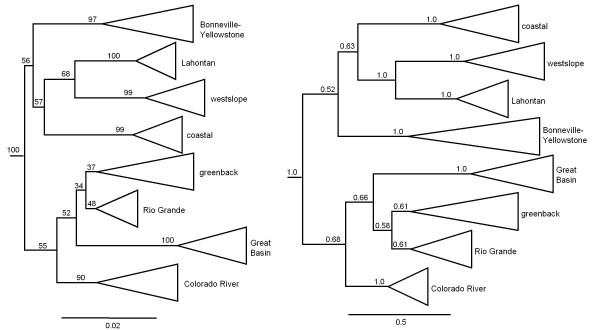
**Phylogenies of cutthroat trout**. **(a) **Maximum likelihood phylogeny of cutthroat trout based on the mitochondrial ND2 gene. Scale bar represents the proportion of sequence divergence in the ND2 gene used to construct the tree. Numbers above branching points represent the percent of bootstrap values for the number of times a node was recovered from 1000 iterations. **(b) **Bayesian phylogeny of cutthroat trout based on the mitochondrial ND2 gene. Numbers indicate Bayesian posterior probabilities. Scale bar represents the number of substitutions per site. In both (a) and (b) the tree was rooted to rainbow trout as the sister taxa, but is not included in the phylogeny for ease of presentation.

Our maximum likelihood analysis identified eight major lineages of cutthroat trout based on mtDNA sequence differences (Figure 3a). Of these eight main groups, four, including coastal, westslope, Lahontan, and Colorado River cutthroat trout, occurred as monophyletic lineages and corresponded to subspecies designations previously used to describe intraspecific variation in cutthroat trout. Two groups (Rio Grande cutthroat trout and greenback cutthroat trout), while forming separate groups in our phylogeny, were not well supported. As indicated in the minimum spanning network, Bonneville and Yellowstone cutthroat trout did not form monophyletic lineages, but were mixed into two well-supported and very divergent clades. The Bonneville-Yellowstone clade included the majority of the sampling locations for these subspecies; however, a distinct and divergent clade is also present in the phylogeny (the Great Basin lineage), and is more closely related to populations from the Colorado River watershed despite its geographic proximity to the upper Snake River and Bear River (Figure 3a). The Paiute and Whitehorse cutthroat trout were nested within the Lahontan branch of the phylogeny (Figure 3a), and fish representing fine-spotted Snake River cutthroat trout had the same haplotype of other Yellowstone cutthroat trout. When we repeated the phylogenetic analysis using a Bayesian approach, we found the arrangement of branches with similar or greater support based on posterior probabilities (Figure 3b).

When we compared the variability in sequence data of cutthroat trout, we found that a predominant proportion was accounted for by differences between the major clades identified. Among the eight major clades, 71.84% of the variability in sequence data was accounted for by differences among clades; whereas 28.16% was accounted for by differences within clades (AMOVA, *P *< 0.0001). Excluding the four unique haplotypes deposited in GenBank for greenback cutthroat trout, haplotype diversity was highest in Bonneville-Yellowstone cutthroat trout and lowest in the westslope cutthroat trout; while nucleotide diversity was also highest in the Bonneville-Yellowstone group, it was lowest in Lahontan cutthroat trout group (Table 1) Across all major cutthroat clades, average sequence divergence was 2.4% (range: 1.3-3.2; Table 2). Average divergence of cutthroat trout lineages with rainbow trout was 8% (range: 7.3-8.7%; Table 2).

**Table 2:** Pairwise percent sequence divergence of the ND2 gene between major cutthroat trout phylogenetic clades and rainbow trout

Taxa/Clade	rainbow	coastal	westslope	Bonneville-Yellowstone	Lahontan	Great Basin	greenback	Colorado River	Rio Grande
rainbow	0								
coastal	8.0	0							
westslope	8.3	2.3	0						
Bonneville-Yellowstone	8.2	2.2	2.7	0					
Lahontan	8.1	2.0	1.9	2.7	0				
Great Basin	8.7	3.0	3.1	1.9	3.2	0			
greenback	7.7	3.0	2.9	3.1	2.5	2.0	0		
Colorado River	8.1	2.2	2.5	2.9	1.9	2.2	1.8	0	
Rio Grande	7.3	2.4	2.6	2.5	2.2	1.8	1.3	1.8	0

### Geographic sub-structuring

**Figure 4:**
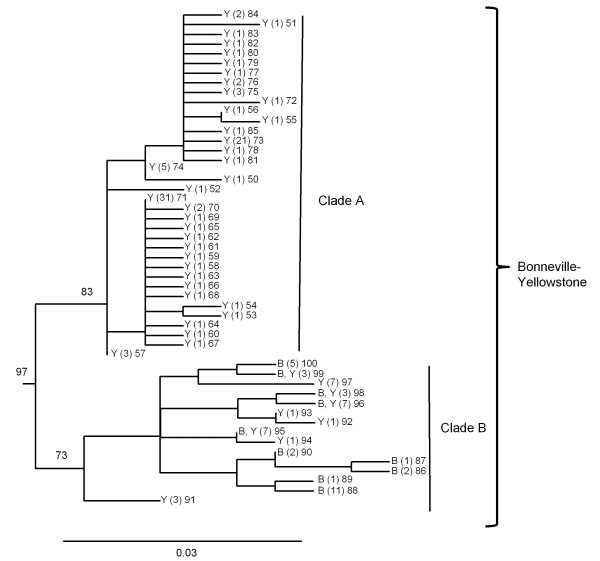
**Phylogeny of Bonneville-Yellowstone cutthroat trout**. Detailed maximum likelihood phylogeny of the Bonneville-Yellowstone lineage of cutthroat trout. Branch tips labeled as Y and B represent haplotypes sampled within the historical distribution of Yellowstone cutthroat trout and Bonneville cutthroat trout, respectively (see Figures 1 and 5). Numbers in parentheses represent the number of sampling locations where the haplotype was detected followed by the haplotype number. Numbers on branches indicate percent bootstrap support based on 1000 replicates. Scale bar represents the proportion of sequence divergence in the ND2 gene used to construct the phylogeny.

**Figure 5:**
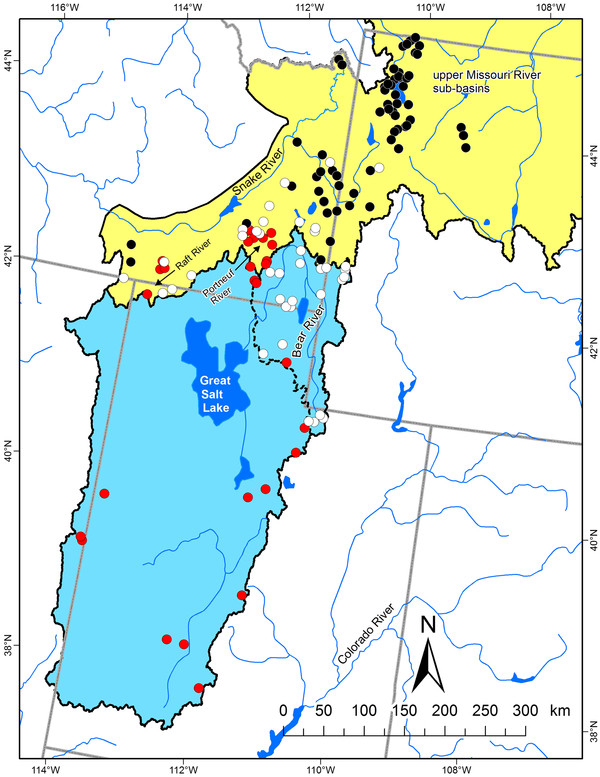
**Geographic distribution of Bonneville-Yellowstone and Great Basin cutthroat trout lineages**. Geographic distribution of two major subclade lineages of Bonneville-Yellowstone cutthroat trout (see Figure 4) depicted by black circles (clade A) and white circles (clade B). Red circles represent sampling locations for the clade of Great Basin cutthroat trout (see Figure 8). Colored polygons represent the estimated boundary for the native range of Bonneville (blue) and Yellowstone (Yellow) subspecies of cutthroat trout [24]. The dashed line represents the extent of the Bear River watershed, excluding the Malad River sub-basin.

**Figure 6:**
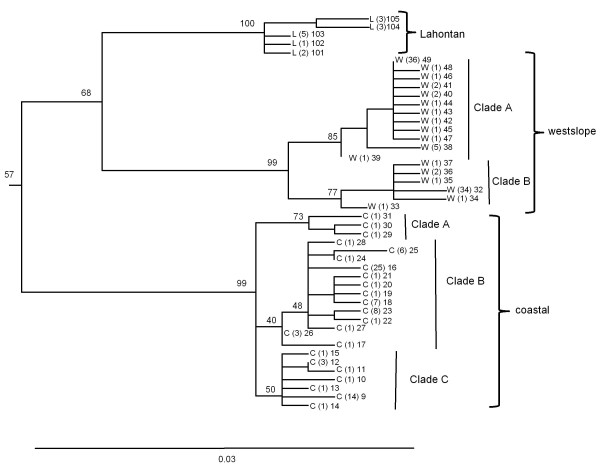
**Phylogeny of Lahontan, westslope, and coastal cutthroat trout**. Detailed maximum likelihood phylogeny of the Lahontan, westslope, and coastal lineages of cutthroat trout. Branch tips labeled as L represent haplotypes sampled within the historical distribution of Lahontan cutthroat trout; whereas those labeled as W and C represent haplotypes sampled within the range of westslope and coastal cutthroat trout, respectively (see Figures 1 and 7 for geographic ranges). Numbers in parentheses represent the number of sampling locations where the haplotype was detected followed by the haplotype number. Numbers on branches indicate percent bootstrap support based on 1000 replicates. Scale bar represents the proportion of sequence divergence in the ND2 gene used to construct the phylogeny.

**Figure 7:**
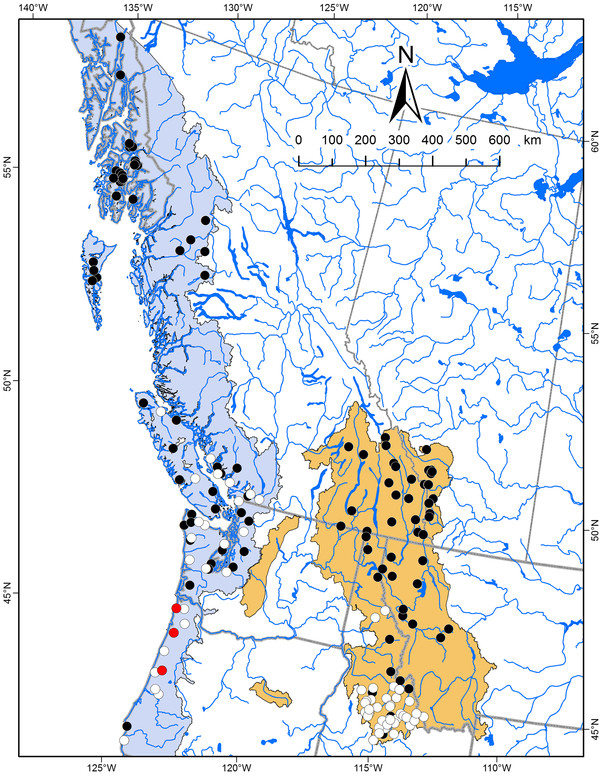
**Geographic distribution of coastal and westslope cutthroat trout lineages**. Geographic distribution of three major subclade lineages of coastal cutthroat trout (see Figure 6) depicted by red circles (clade A), white circles (clade B), and black circles (clade C); and for two major subclade lineages of westslope cutthroat trout depicted by white circles (clade A) and black circles (clade B). Colored polygons represent the estimates extent of the native range of coastal (blue) and westslope (brown) cutthroat trout [24,64].

**Figure 8:**
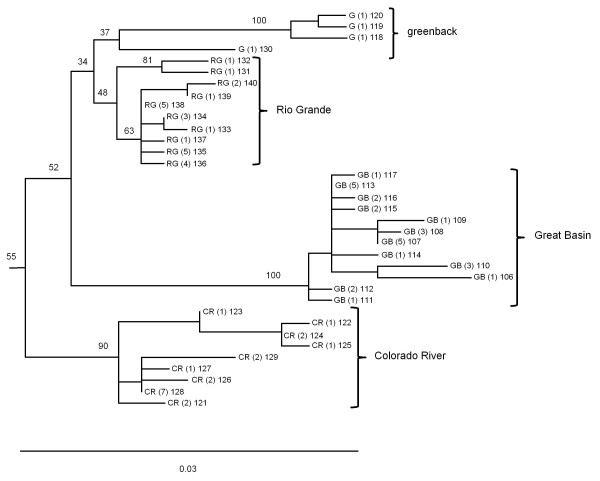
**Phylogeny of greenback, Rio Grande, Great Basin, and Colorado River cutthroat trout lineages**. Detailed maximum likelihood phylogeny of the greenback, Rio Grande, Great Basin, and Colorado River lineages of cutthroat trout. Branch tips labeled as G, RG, and CR represent haplotypes sampled within the historical distribution of greenback, Rio Grande, and Colorado River cutthroat trout; whereas those labeled as GB represent haplotypes sampled within the range of Bonneville or Yellowstone cutthroat trout, respectively (see Figures 1 and 5 for geographic ranges). Numbers in parentheses represent the number of sampling locations where the haplotype was detected followed by the haplotype number. Numbers on branches indicate percent bootstrap support based on 1000 replicates. Scale bar represents the proportion of sequence divergence in the ND2 gene used to construct the phylogeny.

Within several of the major clades of cutthroat trout, there was further geographically related sub-structuring among haplotypes. The Bonneville-Yellowstone clade, which included two subspecies of cutthroat trout, formed two primary subclades (Figure 4). Within this group, clade B included a mixture of haplotypes collected from geographic locations in the Bonneville and upper Snake River watersheds. In contrast, haplotypes from the clade A group were only found in the upper Snake River and upper Missouri River watersheds (Figure 5). Within the coastal cutthroat trout lineage three primary subdivisions were identified (Figure 6). All three were found in areas below the southern end of Vancouver Island, British Columbia, but only haplotypes associated with one of the subdivisions were found in more northern latitudes of British Columbia and southeast Alaska (Figure 7). The westslope cutthroat trout lineage also had two major subdivisions (Figure 6). Both were found in the southern portions of its range in Idaho, but only haplotypes associated with one of the lineages were found in more northerly areas of Montana, British Columbia, and Alberta (Figure 7). Of the five different haplotypes observed in the Lahontan lineage of cutthroat trout, there was no large divergence among the geographic samples collected. The Whitehorse population did have a unique haplotype that was slightly different than populations from the Lahontan basin; whereas the Paiute cutthroat populations all had the same haplotype that was also found in the Humboldt and Reese River systems of Nevada (Additional file 1: Table S1). Within the Colorado River lineage we detected no well supported sub-structuring; haplotypes were distributed throughout the historic range of this subspecies (Additional file 1: Tables S1 and Additional file 2: Table S2). Some of the GenBank sequences also identified the Colorado River lineage across the Continental Divide into the range of greenback cutthroat trout [41]; and one greenback cutthroat trout was found east of the Continental Divide in the Colorado River range. As Metcalf and collaborators [41] note, the greatest divergence in greenback cutthroat trout occurs between Bear Creek, Colorado and the three remaining unique haplotypes (Figure 8). Similar to the findings of Pritchard and others [42], the Rio Grande lineage had two unique haplotypes that were most different from all other haplotypes (Figure 8), one of the two was found in the upper Canadian River watershed of New Mexico (Ricardo Creek), and one in Dalton Creek, a stream in the Pecos River watershed. All other haplotypes were found in the Rio Grande watershed. Within the Great Basin lineage only minor differences in sequence divergence were evident (Figure 8).

### Divergence times

Results of the relative rate test indicated that we were able to apply a molecular clock to our data. Thus, based on the assumption of 6 million years between rainbow trout and cutthroat trout divergence, we estimated that all cutthroat trout shared a common ancestor approximately 1.97 mya, when there was a primary divergence between the lineage that includes the Bonneville-Yellowstone, Lahontan, westslope, and coastal groups versus the lineage including the greenback, Rio Grande, Great Basin, and Colorado River groups. Within each of these major cutthroat trout clades, the Bonneville-Yellowstone lineage was estimated to have diverged from other members of its group at 1.9 mya, followed by the coastal group at 1.85 mya, then between the westslope and Lahontan clades about 1.43 mya (Figure 3a). The Colorado River lineage of cutthroat trout was estimated to have diverged within its subclade at 1.41 mya, followed by the Great Basin lineage at 1.38 mya, and finally between the greenback and Rio Grande lineages at 0.94 mya (Figure 3a).

## Discussion

In this study we examined how cutthroat trout diversity may be organized by isolation into major geographic boundaries. Our data indicate that watershed boundaries do organize primary phylogenetic divisions in cutthroat trout, but historical connections that are no longer evident also appear to have influenced the pattern of diversification. Our range-wide analysis of cutthroat trout diversification provides an additional line of evidence for historic watershed connections that have long been suggested by geologists and are of interest to biologists as a means of explaining the current distribution of fish fauna across a landscape [8,16,18,43].

Intraspecific taxonomic evaluations of cutthroat trout have described up to 14 different subspecies of cutthroat trout, two of which are now considered to be extinct [20,24]. Of the subspecies we evaluated, the Bonneville-Yellowstone, Colorado River, Rio Grande, greenback, and Lahontan lineages are delimited at least in part by the height of land defining major portions of watersheds. Based on the mtDNA sequence data we compared and the partitioning of genetic variance, cutthroat trout can be divided into eight major lineages that correspond with six of the primary subspecies of cutthroat trout commonly recognized. Hence, watershed boundaries and the intraspecific taxonomic categories that have been used to describe this diversity have been relatively successful in capturing a significant component of the evolutionary diversification in cutthroat trout.

If a molecular clock is applied to the sequence divergence, we estimate an initial divergence from a common ancestor approximately 2 mya. This primary divergence led to one major clade that includes the Bonneville-Yellowstone, Lahontan, westslope, and coastal lineages. The second branch includes the greenback, Rio Grande, Great Basin, and Colorado River lineages. The pattern of haplotype diversification indicates that the lineage leading to the Bonneville-Yellowstone clade was first to diverge from the common ancestor of all cutthroat trout, which may have first colonized the ancestral Snake River as well as areas that are now part of Colorado River watershed. Although the pattern of colonization can never be known with exact certainty, drainage patterns have changed significantly over the period of time estimated from the divergence of the ancestral cutthroat trout. The greatest divergence of cutthroat trout between the Snake and Colorado River drainages indicates a former connection between the two drainages that has since isolated these two main lineages for the longest period of time. Geologic evidence does point to connections between areas that are now in the upper Colorado River watershed but were once part of the Bear River of Idaho, Utah, and Wyoming [16].

Within each of the main lineages of cutthroat trout, the pattern of relatedness also indicates how several sub-basins may have been connected to each other. The next closest associations within the Bonneville-Yellowstone group are those from coastal cutthroat trout populations followed by a split between populations from the Lahontan basin (and adjacent areas) and westslope cutthroat trout from the Columbia River as well as populations from eastern drainages across the Continental Divide. Downstream colonization and origin of coastal cutthroat trout could have occurred if the ancestral Snake River was connected to the Columbia watershed or other rivers draining to the Pacific Ocean, but the association of the coastal cutthroat trout with westslope cutthroat trout found in Columbia drainages suggests that the Snake River was part of the Columbia watershed when the lineages emerged. Despite the proximity of some parts of the Lahontan basin to areas of the Colorado River watershed, Hubbs and Miller [43] noted the similarity of the Lahontan fish fauna with that of Columbia, Sacramento, and Death Valley watersheds, indicating that the Lahontan basin was at one time connected to one of these systems and not the Colorado River watershed. Our sequence data on cutthroat trout again point to a connection with the Columbia watershed based on similarity of lineages also found between the watersheds. Finally, the associations of the greenback, Rio Grande, and Great Basin lineages with that of the Colorado lineage all indicate that they were derived in an earlier form of the Colorado River basin. Based on the evolutionary history of the ND2 gene, our data indicate a complex history of isolation and evolutionary divergence of the ancestral Colorado lineage of cutthroat trout. This divergence led to the current Colorado lineage as well as the Rio Grande and Great Basin lineages. The lineage that led to the Rio Grande also appears to have given rise to the greenback lineage, which subsequently colonized its current distribution. The presence of greenback cutthroat trout in eastern draining rivers, or rivers draining to the south in the case of Rio Grande cutthroat, indicate transfers of populations through headwater connections and subsequent isolation. As Minckley and others [16] note, fish species that commonly penetrate into headwater streams often have representative populations across drainage divides presumably by finding connections, even if they occur infrequently, and are further isolated over longer time periods by the elevation of land.

Two subspecies that did not form monophyletic lineages were the Bonneville and Yellowstone cutthroat trout. The core distribution of Bonneville cutthroat trout is thought to occur in the Bear River, a watershed that originates in the Uinta Mountains of northeastern Utah and flows northward into Wyoming and Idaho before turning sharply back southward towards Utah and the Great Salt Lake (Figure 5). Such a dramatic change of direction is probably representative of stream capture, and in fact the Bear River is thought to have been a tributary to the upper Snake River [44]. Between 600 000 and 50 000 years ago, lava flows crossed the Soda Springs area of the Bear River valley and forced the path of the river to change direction southward [44]. A past connection between the Bear River and upper Snake River would explain the intermixing of the primary lineages of cutthroat trout found in these two watersheds, as well as the morphological similarities observed between these two subspecies [24,45]. The presence of the two main cutthroat trout lineages in the upper Snake River, but only one in the Bear River would suggest that it originated in the Bear River and subsequently colonized downstream to the Snake River, whereas the second lineage was unable to colonize upstream to the Bear River.

A new perspective generated from our range wide analysis was the geographic distribution and position of what we have referred to as the Great Basin clade of cutthroat trout. Although some previous studies have detected and noted the dramatic genetic divergence of representative samples from this lineage [27,39], our study provides a clearer picture of its geographic distribution and its position within the phylogeny of cutthroat trout. Our data indicate that despite its proximity to the Bear River and upper Snake River watersheds, it is more closely related to the major diversification of cutthroat trout that includes the Colorado River, greenback, and Rio Grande clades of cutthroat trout. Such a relationship again points to historic connections between watersheds now isolated from each other. As Smith and collaborators [39] note, previous studies have documented possible hydrological connections between the Bonneville basin and the Colorado River watershed. Connections between these watersheds would be congruent with the Great Basin lineage of cutthroat trout arising in an ancient Colorado River area that either invaded the Bonneville basin by a past connection or evolved within the basin when some parts drained toward the Colorado River watershed. The presence of the Great Basin cutthroat lineage in the upper Snake River may also represent past hydrological connections between the Snake River and the Bonneville basin, but were probably more recent, corresponding to well-documented Pleistocene connections [44]. Approximately 15 000 years ago, Red Rock pass in southeast Idaho was the path of overflow of Lake Bonneville into the upper Snake River travelling through upper Marsh Creek and the Portneuf River; the same location where we detected the Great Basin cutthroat trout lineage [46]. The second location where we observed the Great Basin lineage in the upper Snake River watershed was in the Raft River drainage in southern Idaho, about 150 km to the west of the Portneuf River (Figure 5). This area borders the Bonneville basin and our data suggest that part of the current Raft River drainage was previously in the Bonneville basin and has been captured by the Snake River watershed, isolating the Great Basin cutthroat trout in the headwater tributary streams observed today. Such captures may not be too unexpected because of the gradual subsidence of the Snake River plain that may capture streams bordering the region [47]. As noted in phylogeographic studies of galaxiid fish in New Zealand, geographic patterns of genetic diversity can provide an additional line of evidence to infer historical changes to drainage patterns on the landscape [48].

Watershed boundaries and barriers to movement can organize major phylogenetic lineages for freshwater fishes in some cases but appear to be most important when populations have been isolated within them for extended periods of time. Glaciated areas often appear to have been more recently connected, allowing major lineages to disperse from glacial refugia over large geographic areas, as illustrated by examples of lake trout (*Salvelinus namaycush*) and lake whitefish (*Coregonus clupeaformis*). Each of these species is thought to have dispersed through large proglacial lakes that submerged or connected a number of contemporary watersheds in North America [49,50]. Populations of freshwater fish that have not established themselves by dispersal into recently de-glaciated areas appear much more likely to exhibit significantly greater evolutionary diversification as a consequence of isolation within watershed boundaries [13,15,51]. As a widely distributed species spanning both glaciated and non-glaciated regions of North America, cutthroat trout exhibit reduced haplotype diversity at the northern periphery of its range and higher levels to the south. A reduction in genetic diversity in northern regions is concordant with a pattern of dispersal from a past southern glacial refuge, illustrating the importance of past geologic events on the phylogenetic structure of a freshwater fish species [17].

As a widely distributed trout species in North America, cutthroat trout have received a great deal of interest as a sport fish, but have also suffered severe declines in abundance when populations have been affected by human plans for consumption of water, land, or introductions of non-native species (see Trotter [19] for a review). Despite early and more recent attempts to describe the diversity of cutthroat trout [21,24] it is somewhat surprising that there is remarkably little comparative data to support subspecific designations commonly used to manage different cutthroat trout populations [52]. Perhaps as a result of a lack of such data, only one taxonomic ranking of cutthroat trout is currently recognized by the joint Committee on Names of Fishes, sponsored by the American Fisheries Society and the American Society of Ichthyologists and Herpetologists [53], and possibly because of the difficulty in diagnosing subspecies of cutthroat trout with traditional meristic characters [54]. A number of previous studies have provided evidence for significant genetic diversification of cutthroat trout [55-58] and even phylogenetic comparisons of representative populations from different parts of the species range [27,39,59]. However, our study provides the first range-wide comparison attempting to identify major phylogenetic lineages over much of the geographic area occupied by this polytypic species.

Our analysis provides a better understanding of the main evolutionary lineages of cutthroat trout and their geographic distribution. Similar components have been proposed as a basis for organizing management and conservation units of species [3,60]. Although primary evolutionary lineages of cutthroat trout may be a logical starting point for management units, even with the addition of genetic diversity at nuclear loci, they may not capture important ecological variation that can occur has as result of local adaption to specific conditions [61,62]. Hence, in addition to measures of mtDNA and nuclear DNA divergence, ecological diversification not always apparent from molecular phylogenies should also be considered in attempts to preserve the diversity present within a species. Indeed, salmonid fishes often exhibit significant ecological variation [63] and cutthroat trout in particular have been observed to exhibit significant morphological variability that is associated with specific ecological conditions [45]. Fortunately, biologists have often used the precautionary principle in arguing for protecting populations with unusual life history or ecological relationships even in the absence of direct comparative data. By combining comprehensive range-wide phylogenetic comparisons to identify the distribution of major species lineages, with an understanding of the main ecological factors shaping the phenotype of a species, biologists are likely to provide the best chance of protecting the evolutionary potential of a species in a changing landscape.

## Conclusions

Cutthroat trout populations have diversified into major phylogenetic lineages in western North America. While geographic isolation within major watershed boundaries is a primary organizing factor of genetic diversity in cutthroat trout, past hydrological connections have also influenced the evolutionary history of cutthroat trout. Our study illustrates how genetic diversity can be used to identify where historic watershed connections may have existed and how that information can help explain the current distribution of biological diversity across a landscape.

## Authors' contributions

Both Authors fully participated in the design, collection of specimens, data analysis, and writing of the manuscript. Both authors have read and approved the final manuscript.

## Supplementary Material

Additional file 1 **Table S1**. Population, phylogenetic grouping, geographic location, and accession number of haplotypes for trout sampled in this study. Click here for file

Additional file 2 **Table S2**. Phylogenetic clade, haplotype, and accession number for trout used from Genbank database. Click here for file

## References

[B1] Schluter D The ecology of adaptive radiation 2000 Oxford, UK: Oxford University Press

[B2] Via S The ecological genetics of speciation Am Nat 2002 159 S1 S7 10.1086/338368 18707366

[B3] Crandall KA Bininda-Emonds ORP Mace GM Wayne RK Considering evolutionary processes in conservation biology Trends Ecol Evol 2000 15 290 295 10.1016/S0169-5347(00)01876-0 10856956

[B4] Moritz C Strategies to protect biological diversity and the evolutionary processes that sustain it Syst Biol 2002 51 238 254 10.1080/10635150252899752 12028731

[B5] Sexton J McIntyre P Angert A Rice K Evolution and ecology of species range limits Annu Rev Ecol Syst 2009 40 415 436 10.1146/annurev.ecolsys.110308.120317

[B6] Lavergne S Mouquet N Thuiller W Ronce O Biodiversity and climate change: integrating evolutionary and ecological responses of species and communities Annu Rev Ecol Syst 2010 41 321 350 10.1146/annurev-ecolsys-102209-144628

[B7] Hubbs CL Miller RR Hubbs LC Hydrographic history and relict fishes of the north-central Great Basin 1974 San Francisco, CA: California Academy of Sciences

[B8] McPhail JD Lindsey CC Hocutt CH, Wiley EO Zoogeography of the freshwater fishes of Cascadia (the Columbia system and rivers north to the Stikine) The zoogeography of North American freshwater fishes 1986 New York, NY: Wiley-Interscience 615 637

[B9] Avise JC Arnold J Ball RM Bermingham E Lamb T Neigel JE Reeb CA Saunders NC Intraspecific phylogeography: the mitochondrial DNA bridge between population genetics and systematics Annu Rev Ecol Syst 1987 18 489 522

[B10] Pérez-Espona S Pérez-Barbería FJ McLeod JE Jiggins CD Gordon IJ Pembeton JM Landscape features affect gene flow of Scottish Highland red deer (*Cervus elaphus*) Mol Ecol 2008 17 981 996 10.1111/j.1365-294X.2007.03629.x 18261043

[B11] Loxterman JL Fine scale population genetic structure of pumas in the Intermountain West Conserv Genet 2011 12 1049 1059 10.1007/s10592-011-0208-y

[B12] Taylor EB Stamford MD Baxter JS Population subdivision in westslope cutthroat trout (*Oncorhynchus clarki lewisi*) at the northern periphery of its range: evolutionary inferences and conservation implications Mol Ecol 2003 12 2609 2622 10.1046/j.1365-294X.2003.01937.x 12969465

[B13] Pfrender M Hicks J Lynch M Biogeographic patterns and current distribution of molecular-genetic variation among populations of speckled dace, *Rhinichthys osculus *(Girard) Mol Phylogenet Evol 2004 30 490 502 10.1016/S1055-7903(03)00242-2 15012934

[B14] Gomez-Uchida D Knight TW Ruzzante DE Interaction of landscape and life history attributes on genetic diversity, neutral divergence and gene flow in a pristine community of salmonids Mol Ecol 2009 18 4854 4869 10.1111/j.1365-294X.2009.04409.x 19878451

[B15] Faulks LK Gilligan DM Beheregaray LB Islands of water in a sea of dry land: hydrological regime predicts genetic diversity and dispersal in a widespread fish from Australia's arid zone, the golden perch (*Macquaria ambigua*) Mol Ecol 2010 19 4723 4737 10.1111/j.1365-294X.2010.04848.x 20887362

[B16] Minckley WL Hendrickson DA Bond CE Hocutt CH, Wiley EO Geography of western North American freshwater fishes: description and relationships to transcontinental tectonism The zoogeography of North American freshwater fishes 1986 New York, NY: Wiley-Interscience 519 613

[B17] Bernatchez L Wilson CC Comparative phylogeography of nearctic and palearctic fishes Mol Ecol 1998 7 431 452 10.1046/j.1365-294x.1998.00319.x

[B18] Waters JM Craw D Youngson JH Wallis GP Genes meet geology: fish phylogeographic pattern reflects ancient, rather than modern, drainage connections Evolution 2001 55 1844 1851 11681739 10.1111/j.0014-3820.2001.tb00833.x

[B19] Behnke RJ Trout and salmon of North America 2002 New York, NY: Free Press

[B20] Trotter PC Cutthroat: native trout of the west 2008 2 Berkeley, CA: University of California Press

[B21] Jordan DS Evermann BW Clark HW Check list of the fishes and fishlike vertebrates of North and Middle America north of the northern boundary of Venezuela and Colombia 1930 Washington, DC: U.S. Govt. Print. Off

[B22] Pielou EC After the ice age: the return of life to glaciated North America 1991 Chicago, IL: University of Chicago Press

[B23] Young MK Conservation assessment for inland cutthroat trout 1995 Fort Collins, CO: U.S: Department of Agriculture, Forest Service, Rocky Mountain Research Station

[B24] Behnke RJ Native trout of western North America 1992 Bethesda, MD: American Fisheries Society

[B25] Trotter PC Behnke RJ The case for *humboldtensis*: a subspecies name for the indigenous cutthroat trout (*Oncorhynchus clarkii*) of the Humboldt River, Upper Quinn River, and Coyote Basin drainages, Nevada and Oregon West N Am Naturalist 2008 68 58 65 10.3398/1527-0904(2008)68[58:TCFHAS]2.0.CO;2

[B26] Novak M Mock K Kershner J Molecular genetic investigation of Yellowstone cutthroat trout and finespotted Snake River cutthroat trout 2005 Logan, UT: Utah State University

[B27] Campbell MR Kozfkay CC Meyer KA Powell MS Williams RN Historical influences of volcanism and glaciation in shaping mitochondrial DNA variation and distribution in Yellowstone cutthroat trout across its native range Trans Amer Fish Soc 2011 140 91 107

[B28] Edgar RC MUSCLE: multiple sequence alignment with high accuracy and high throughput Nucleic Acids Res 2004 32 1792 1797 10.1093/nar/gkh340 15034147 PMC390337

[B29] Excoffier L Lischer HEL Arlequin suite ver 3.5: a new series of programs to perform population genetics analyses under Linux and Windows Mol Ecol Resour 2010 10 564 567 10.1111/j.1755-0998.2010.02847.x 21565059

[B30] Librado P Rozas J DnaSP v5: a software for comprehensive analysis of DNA polymorphism data Bioinformatics 2009 25 1451 1452 10.1093/bioinformatics/btp187 19346325

[B31] Tamura K Peterson D Peterson N Stecher G Nei M Kumar S MEGA5: molecular evolutionary genetics analysis using maximum likelihood, evolutionary distance, and maximum parsimony methods Mol Biol Evol 2011 28 2731 2739 10.1093/molbev/msr121 21546353 PMC3203626

[B32] Posada D jModelTest: Phylogenetic Model Averaging Mol Biol Evol 2008 25 1253 1256 10.1093/molbev/msn083 18397919

[B33] Guidon S Dufayard J-F Lefort V Anisimova M Hordijk W Gascuel O New Algorithms and Methods to Estimate Maximum-Likelihood Phylogenies: Assessing the Performance of PhyML 3.0 Syst Biol 2010 59 307 321 10.1093/sysbio/syq010 20525638

[B34] Huelsenbeck JP Ronquist F MRBAYES: Bayesian inference of phylogenetic trees Bioinformatics 2001 17 754 755 10.1093/bioinformatics/17.8.754 11524383

[B35] Ronquist F Huelsenbeck JP MrBayes 3: Bayesian phylogenetic inference under mixed models Bioinformatics 2003 19 1572 1574 10.1093/bioinformatics/btg180 12912839

[B36] Nylander JAA MrModeltest v2 2004 Uppsala University: Program distributed by the author. Evolutionary Biology Centre

[B37] Tajima F Simple methods for testing the molecular evolutionary clock hypothesis Genetics 1993 135 599 607 8244016 10.1093/genetics/135.2.599 PMC1205659

[B38] McKay SJ Devlin RH Smith MJ Phylogeny of Pacific salmon and trout based on growth hormone type-2 and mitochondrial NADH dehydrogenase subunit 3 DNA sequences Can J Fish Aquat Sci 1996 53 1165 1176 10.1139/f96-042

[B39] Smith GR Dowling TE Goblat KW Lugaski T Shiozawa DK Evans RP Hershler R, Madsen D, Currey DR Biogeography and timing of evolutionary events among Great Basin fishes Great Basin aquatic systems history 2002 Washington, DC: Smithsonian Institution Press 175 254

[B40] Janetski DJ Genetic considerations for the conservation and management of Yellowstone cutthroat trout (Oncorhynchus clarkii bouvieri) in Yellowstone National Park 2006 Provo UT: Thesis, Brigham Young University

[B41] Metcalf JL Pritchard VL Silvestri SM Jenkins JB Wood JS Cowley DE Evans RP Shiozawa DK Martin AP Across the great divide: genetic forensics reveals misidentification of endangered cutthroat trout populations Mol Ecol 2007 16 4445 4454 10.1111/j.1365-294X.2007.03472.x 17727621

[B42] Pritchard VL Metcalf JL Jones K Martin AP Cowley DE Population structure and genetic management of Rio Grande cutthroat trout (*Oncorhynchus clarkii virginalis*) Conserv Genet 2009 10 1209 1221 10.1007/s10592-008-9652-8

[B43] Hubbs CL Miller RR The zoological evidence: correlation between fish distribution and hydrographic history in the desert basins of western United States The Great Basin, with an emphasis on postglacial times 1948 38 Salt Lake, UT: University of Utah 17 166

[B44] Link PK Kaufman DS Thackray GD Hughes SS, Thackray GD Field guide to Pleistocene lakes Thatcher, and Bonneville and the Bonneville Flood, southeastern Idaho Guidebook to the geology of eastern Idaho 1999 Pocatello, ID: Idaho Museum of Natural History 251 266

[B45] Seiler MB Keeley ER Intraspecific taxonomy and ecology characterize morphological divergence among cutthroat trout (*Oncorhynchus clarkii *ssp Richardson) populations Biol J Linn Soc 2009 96 266 281 10.1111/j.1095-8312.2008.01130.x

[B46] Oviatt CG Currey DR Sack D Radiocarbon chronology of Lake Bonneville, Eastern Great Basin, USA Palaeogeogr Palaeocl Palaeoecol 1992 99 225 241 10.1016/0031-0182(92)90017-Y

[B47] Pierce KL Morgan LA Link PK, Kuntz MA, Platt LB The track of the Yellowstone hot spot: volcanism, faulting, and uplift Regional geology of eastern Idaho and Western Wyoming 1992 Geological Society of America 1 52

[B48] Waters JM Allibone RM Wallis GP Geological subsidence, river capture, and cladogenesis of galaxiid fish lineages in central New Zealand Biol J Linnean Soc 2006 88 367 376 10.1111/j.1095-8312.2004.00622.x

[B49] Bernatchez L Dodson JJ Phylogeographic structure in mitochondrial DNA of the lake whitefish (*Coregonus clupeaformis*) and its relation to Pleistocene glaciations Evolution 1991 45 1016 1035 10.2307/2409706 28564052

[B50] Wilson CC Hebert PDN Phylogeography and postglacial dispersal of lake trout (*Salvelinus namaycush*) in North America Can J Fish Aquat Sci 1998 55 1010 1024 10.1139/f97-286

[B51] Bermingham E Avise JC Molecular zoogeography of freshwater fishes in the southeastern United States Genetics 1986 113 939 965 17246340 10.1093/genetics/113.4.939 PMC1202920

[B52] Gresswell RE Status and management of interior stocks of cutthroat trout 1988 Bethesda, MD: American Fisheries Society Symposium 4

[B53] Nelson JS Crossman EJ Espinosa-Pérez H Findley L Gilbert CR Lea RN Williams JD Common and scientific names of fishes from the United States Canada and Mexico 2004 6 Bethesda, MD: American Fisheries Society

[B54] Allendorf FW Leary RE Hitt NP Knudsen KL Boyer MC Spruell P Cutthroat trout hybridization and the U.S. Endangered Species Act: one species, two policies Conserv Biol 2005 19 1326 1328 10.1111/j.1523-1739.2005.00223.x

[B55] Klar GT Stalnaker CB Electrophoretic variation in muscle lactate dehydrogenase in Snake Valley cutthroat trout,*Salmo clarki *subsp Comp Biochem Physiol, B 1979 64 391 394 10.1016/0305-0491(79)90288-8 318316

[B56] Loudenslager EJ Gall GAE Geographic patterns of protein variation and subspeciation in cutthroat trout, *Salmo clarki* Syst Zool 1980 29 27 42 10.2307/2412624

[B57] Martin MA Shiozawa D Loudenslager EJ Jensen JN Electrophoretic study of cutthroat trout populations in Utah West N Am Naturalist 1985 45 677 687

[B58] Leary RF Allendorf FW Phelps SR Knudsen KL Genetic divergence and identification of seven cutthroat trout subspecies and rainbow trout Trans Amer Fish Soc 1987 116 580 587 10.1577/1548-8659(1987)116<580:GDAIOS>2.0.CO;2

[B59] Wilson WD Turner TF Phylogenetic analysis of the Pacific cutthroat trout (*Oncorhynchus clarki *ssp.: Salmonidae) based on partial mtDNA ND4 sequences: a closer look at the highly fragmented inland species Mol Phylogenet Evol 2009 52 406 415 10.1016/j.ympev.2009.03.018 19341807

[B60] Bernatchez L Nielsen JL A role for molecular systematics in defining evolutionarily significant units in fishes Evolution and the aquatic ecosystem: defining unique units in population conservation 1995 Bethesda, MD: American Fisheries Society 114 132

[B61] Ford MJ Hendry AP, Stearns SC Conservation units and preserving diversity Evolution illuminated: salmon and their relatives 2004 New York, NY: Oxford University Press 338 357

[B62] Taylor EB Tamkee P Keeley ER Parkinson EA Conservation prioritization in widespread species: the use of genetic and morphological data to assess population distinctiveness in rainbow trout (*Oncorhynchus mykiss*) from British Columbia, Canada Evol Appl 2011 4 100 115 10.1111/j.1752-4571.2010.00136.x PMC3352517 25567956

[B63] Keeley ER Parkinson EA Taylor EB Ecotypic differentiation of native rainbow trout (*Oncorhynchus mykiss*) populations from British Columbia Can J Fish Aquat Sci 2005 62 1523 1539 10.1139/f05-062

[B64] McPhail JD The freshwater fishes of British Columbia 2007 Edmonton, AB: The University of Alberta Press

